# Reply: Modulation of plasma complement by the initial dose of epirubicin/docetaxel therapy in breast cancer and its predictive value

**DOI:** 10.1038/sj.bjc.6606069

**Published:** 2011-01-11

**Authors:** A Michlmayr, T Bachleitner-Hofmann, M Bergmann, R Oehler

**Affiliations:** 1 Department of Surgery, Medical University of Vienna, Anna Spiegel Forschungsgebäude 25.05.003, Waehringer Guertel 18-20, Vienna A-1090, Austria

**Sir,**

We appreciate the comments provided by Garantziotis (2011) on our recent publication in *British Journal of Cancer* (Michlmayr *et al*, 2010). In this study, we investigated whether the protein expression profile in plasma samples from breast cancer patients changes within a few days in response to the initial dose of epirubicin/docetaxel therapy. The expression of several plasma proteins was found to be modulated by the therapy, including inter-*α*-trypsin inhibitor (I*α*I) and different members of the complement cascade. We focused our attention on the complement components and might have underestimated the potential importance of increased I*α*I levels.

I*α*I proteins are a family of structurally related serine protease inhibitors with hyaluronan-binding capacities, assembled from a light chain and one of five homologous heavy chains (H1–H5). In correspondence, separation of I*α*I by two-dimensional gel electrophoresis results in different spots with a molecular weight of ∼80, 125 and 250 kDa (Josic *et al*, 2006). In our study, we found that the abundance of several 125 kDa I*α*I spots (388, 393, 397, 405, 406 and 407) is influenced by the epirubicin/docetaxel therapy (see Figure 1A). All these spots reacted nearly identical to the treatment. Figure 1B shows the expression level of the I*α*I spot 405 before and after the initial dose of epirubicin/docetaxel (time a and b, respectively). The abundance of this spot increased in nearly all patients (*n*=22; increase=32±28%). Garantziotis referred in his letter to a recently found interaction of I*α*I with the complement system. In an experimental study, he could show that I*α*I attenuates *in vitro* complement activation and reduces *in vivo* complement-induced lung injury (Garantziotis *et al*, 2007). This is of special interest with respect to our findings. Our study revealed that the plasma level of total C3 decreases in response to epirubicin/docetaxel therapy (Figure 1C). Comparing the abundances of I*α*I (spot 405) with the total plasma C3 revealed that both parameters correlate negatively with each other (Spearman's Rho *r*=−0.49, *P*<0.01, *n*=44). The total plasma C3 was measured by a routine nephelometric immunoassay that cannot distinguish between the different C3 isoforms. In our study, we also investigated the single isoforms of C3 by two-dimensional gel electrophoresis. They responded unequally to the therapy and some of them correlated with the tumour size at the end of treatment. However, none of these single isoforms showed a correlation with the I*α*I spots, and no 125 kDa I*α*I spot showed a correlation with the success of therapy.

Although these findings corroborate the interaction of I*α*I and the complement system, several questions remain. Is the therapy-induced increase in plasma I*α*I the causative factor for C3 reduction? Is the decrease in C3 associated with reduced complement reactivity? How do the other I*α*I isoforms (80 and 250 kDa) react in response to therapy (they were not identified on our 2D gels)? Further studies are needed to find satisfactory answers.

## Figures and Tables

**Figure 1:**
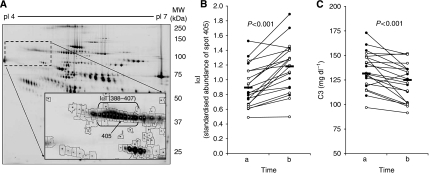
Inter-*α*-trypsin inhibitor (I*α*I) in plasma samples. (**A**) Two-dimensional gel electrophoresis of plasma proteins indicating the 125 kDa I*α*I spots. (**B**) Standardised abundance of spot 405 before (time a) and after (time b) the initial dose of epirubicin/docetaxel given to 22 breast cancer patients. (**C**) Plasma total levels of complement component C3, as determined by immunonephelometry.

## References

[bib1] Garantziotis S (2011) Modulation of plasma complement by the initial dose of epirubicin/docetaxel therapy in breast cancer and its predictive value. Br J Cancer; e-pub ahead of print 11 January 2011, doi: 10.1038/sj.bjc.6606068 10.1038/sj.bjc.6606068 PMC3049565 21224852

[bib2] Garantziotis S, Hollingsworth JW, Ghanayem RB, Timberlake S, Zhuo L, Kimata K, Schwartz DA (2007) Inter-alpha-trypsin inhibitor attenuates complement activation and complement-induced lung injury. J Immunol 179(6): 4187–4192 17785858 10.4049/jimmunol.179.6.4187

[bib3] Josic D, Brown MK, Huang F, Lim YP, Rucevic M, Clifton JG, Hixson DC (2006) Proteomic characterization of inter-alpha inhibitor proteins from human plasma. Proteomics 6(9): 2874–2885 16596706 10.1002/pmic.200500563

[bib4] Michlmayr A, Bachleitner-Hofmann T, Baumann S, Marchetti-Deschmann M, Rech-Weichselbraun I, Burghuber C, Pluschnig U, Bartsch R, Graf A, Greil R, Allmaier G, Steger G, Gnant M, Bergmann M, Oehler R (2010) Modulation of plasma complement by the initial dose of epirubicin/docetaxel therapy in breast cancer and its predictive value. Br J Cancer 103(8): 1201–1208 20877360 10.1038/sj.bjc.6605909 PMC2967072

